# Elements of Medicine. Vol. I. On Morbid Poisons

**Published:** 1838-04

**Authors:** 


### Art. III.

*Elements of Medicine.* Vol. I. *On Morbid Poisons.* By Robert Williams, m.d., Senior Physician of St. Thomas's Hospital.—*London*, 1836. 8vo. pp. 342.

The first volume of this work (the only one yet published) is devoted to the consideration of certain diseases, regarded by its author as both contagious and infectious,—viz. Typhus, Scarlatina, Morbilli, Variolæ, Varicella, Erysipelas, Pertussis. Any one at all conversant with the diversity in the phenomena of these diseases, with the various hypotheses which have prevailed respecting them, and with the different modes of treatment which have been employed in correspondence with such hypotheses, will receive with no little willingness, and perhaps be disposed to scrutinize with too little care, an attempt to explain them consistently with well-known facts, with which they appear to hold the closest relationship. Such is the object of Dr. Williams, as stated in his preface.

"The course, the symptoms, and the pathological phenomena of typhus, as well as of many other diseases depending on the action of a morbid poison, have been determined with so much accuracy, that little hope remains of new facts being added to those already discovered. But, although we are thus in the possession of the necessary data, little progress has been made in deducing a sound theory, or in determining any general rule of treatment in those instances for which no known specific remedy exists. It appearing, however, to be an almost demonstrable truth that, when a given disease could be shewn to depend on the agency of a poison, in whatever manner generated, the laws and treatment of such affection must necessarily follow those of poisons generally, it remained to prove this hypothesis. The result is now submitted to the public; and, if the argument shall be considered as established, it must be considered that contagion is as important an agent in medicine as gravity in mechanics, or electricity in chemistry. The opportunities which a large hospital affords have made it a duty to deviate, in some instances, from the usual routine of practice, and to reduce the treatment to the simplest forms; and the results of these experiments, especially in fever, erysipelas, and scarlet fever, have been so remarkable, that it is submitted, whether at present they ought not to be adopted as the basis of our practice?"

In a carefully written introduction, of which we shall give a brief analysis, Dr. Williams has stated the facts, and some of the reasonings, on which his theory is founded. Others, of course, are derived from the consideration of the diseases with which the volume is occupied; and these diseases we shall mainly consider as illustrative of the hypothesis promulgated, particularly when any mode of treatment is suggested which appears to be more worthy of employment than that generally adopted.

Certain diseases are produced by morbid poisons. The laws governing these do not greatly differ from those of poisons generally. Poisons, of whatever nature, are subjected to certain general laws: they have a *definite specific action*; a *period of latency*; the *phenomena vary according to the dose and predisposition of the individual.* Of the first law, jalap, acting on the mucous membrane of the intestines, is an illustration. Digitalis acts on an organ, the heart; strychnine on a system of organs. More generally, two or more membranes, or organs or systems of organs, are affected; as by elaterium, antimony, opium, &c. Poisons may coexist in the same system, and their actions may be simultaneous on the same tissue: e. g. opium and digitalis, jalap and mercury, produce their respective effects at the same time. Some poisons are cumulative. The *latency of poisons* is evinced by opium, &c. When a medicine acts on more parts than one, a considerable space of time may elapse, after it has affected one organ, before it affects another: thus, mercury affects the bowels for many weeks before its ultimate action on the salivary glands. Oxalic acid and arsenic afford familiar examples of the effect of *dose*. The larger the dose, or the greater the intensity of the poison, the more rapid is its action, and the less the probability of finding any specific trace of disease. Mercury, in its various actions, is a familiar example of the influence of *predisposition*. Habit exercises on some an influence, but not on others. The state of the constitution has also a powerful influence on the action of poisons; a circumstance which needs no illustration, as it occurs to every one in daily practice. Generally, medicines may be said to act with a power proportioned to the debility of the subject. Still, states of disease, though greatly debilitating, render the constitution insusceptible to the action of even powerful remedies; e. g. typhus fever and vinous stimuli, opium and tetanus, &c.

The general laws observable in the actions of morbid poisons are, for the most part, precisely similar to those which govern medicinal substances, or only differ in a few minor points. They have their *specific actions*; *latent periods*, while their phenomena vary with *the dose, and with the predisposition of the patient*. The *specific actions* are shewn by the distinctions between small-pox and ague, ague and syphilis, &c. Like medicinal substances, their actions are variously limited; some affecting only one membrane, or organ, or system of organs; whilst others involve two or more membranes, or organs, or systems of organs. Tinea capitis, bronchocele, hooping-cough, measles, scarlatina, ague, &c. may be mentioned as illustrations of the foregoing statement. Morbid poisons co-exist in the same individual, and even produce their specific effects on the same membrane at the same time. Thus, erysipelas exists with syphilitic eruptions; measles with small-pox; syphilis with hooping-cough, &c. &c. Morbid poisons have also a *latent period*, which requires no illustration. When they act on more tissues or organs than one, their actions are sometimes simultaneous, but more commonly consecutive; and much time often elapses between each successive attack. Typhus and syphilis are examples of this. Poisons, which usually act on a plurality of membranes, sometimes exhaust themselves on one, without affecting the whole series; e. g. rubeola sine catarrho. There appears to be no absolute order in the series of membranes affected by morbid poisons, when more than one is so affected.

The effects of medicinal substances vary with the dose. With morbid poisons, the living body is the only measure which we possess of the strength of the dose. It is impossible to speak of this law, except in conjunction with the temperament or susceptibility of the patient; but, thus considered, the law holds equally good with respect to morbid as to other poisons. There can be no question but that the paludal miasmata of tropical countries greatly exceed in intensity those of more temperate climates; and accordingly, in many cases, hardly a trace of disease is to be found after yellow fever; so intensely severe and rapid is that disorder. But, in the Walcheren fever, a disease perhaps equally fatal, but more chronic and of less intensity, enlarged livers, disorganized spleens, and dropsy marked every case. It may be stated as a law, that, when a morbid poison acts with its greatest intensity and produces its severest forms of disease, fewer traces of organic alteration of structure will be found than when the disorder has been of a milder character. Generally, but there are exceptions, morbid poisons act with an intensity proportioned to the feebleness of the patient.

Such are the chief points of agreement of the laws of morbid poisons with poisons generally. The following are the chief points of difference:—There is no authentic cumulative poison. The quantity of a morbid poison necessary to produce disease is often so small as to be scarcely appreciable. When the quantity is sufficient to produce the specific disease, the result is determined by the temperament or constitution of the patient. The faculty which the body possesses of generating, to an immense extent, a poison of the same nature as that by which the disease was produced, is one wholly unknown to medicinal poisons; as also is that law by which the susceptibility of the constitution, when acted upon by some morbid poisons, becomes exhausted; the body being incapable of again being similarly affected. Lastly, climate or season does not influence the action of vegetable or mineral poisons; but they not only modify the intensity of morbid poisons, but influence also their specific actions. Typhus does not exist at the equator. In different seasons, in this country, it is attended with different structural lesions. It is important to remember, in the study of morbid poisons, that absolute uniformity of pathological phenomena, and consequently of symptoms, is not to be expected. It is sufficient for the purposes of science to have proved the general law, and to have determined the limits within which nature has bounded her deviations.

There is considerable ingenuity in this attempt to demonstrate (as stated in the preface,) "that, where a given disease could be shewn to depend on the agency of a poison, however generated, the laws and treatment of such disease must necessarily follow those of poisons generally;" and the full consideration of the diseases to which this volume is devoted will afford further illustration of the author's views. But we cannot yet agree with Dr. Williams that, granting the existence of morbid poisons, the laws and treatment of them necessarily follow from those of poisons generally. Certain points of resemblance between the effects of one and of the other have been shewn to exist; certain remarkable distinctions have likewise been demonstrated. The question arises, therefore, as to what amount of difference would contradict his conclusion? what degree of similarity is necessary to justify it? On the one hand, if we admit that, in possessing a specific and limited action, a latent period, and an action depending on dose and predisposition, they closely resemble one another; (and, with regard to the third point of similarity, it would be very easy to raise a question;) we must also admit that the protective influence afforded to the constitution by one attack and the peculiarity on which depends the multiplication of the poison to an unlimited amount, constitute very important points of distinction. Until we know whether these differences depend on the poisons themselves, or on the quality of their relations to the body; until, in fact, we know something about them with which we are not at present acquainted, we cannot be justified in the inference, that the same principles of treatment are applicable alike to the diseases produced by both. Instead of applying the term poisons to both, let us call them agents. The question would then stand thus: There are two classes of agents, differing in their chemical constitution, which, when taken into the body, produce certain effects. Some only of these effects resemble each other. It is not, therefore, a legitimate inference that the same laws and treatment are applicable to both. It is begging the whole question to term these minor points of difference. The cause of one of these minor points of difference *may* be that on which depends the appropriateness of this or that system of treatment. We are, therefore, of opinion that any inferential reasoning from poisons generally to morbid poisons, as it regards treatment, is not strictly applicable; but, in other respects, and particularly in the explanation of certain pathological difficulties, the analogy may be applicable, as we shall hereafter see; and not at all the less valuable may be the means recommended by Dr. Williams. We, however, must examine these simply with respect to their results, and as deriving no importance from the reasoning which has led to their employment.

Dr. Williams regards it as proved—(1) that morbid poisons act according to fixed and definite laws, modified only by the influence of climate, temperament, or the magnitude of the dose; (2) that they mingle with the blood, with which they continue in latent combination a certain but very varying time;* (3) that many of them are capable of coexisting in the same system. And two other laws result from the study of morbid poisons,—(1) that they are not acted upon by medicinal substances as long as they continue latent; (2) when they act on more tissues than one,

* The proof of their mingling with the blood is the fact, that some medicinal substances have been detected in that fluid,—e. g. iodine; and that glanders has been communicated, by injecting into the veins of a healthy ass the blood drawn from a glandered horse.

the remedy which is an antidote to their action on one is absolutely powerless when they affect another tissue, so that many different remedies are frequently necessary to combat the varying phenomena of the same disease.

We have a great objection to the frequent application of the term *law* to questionable doctrines in medicine, (and how few are not questionable?) and we can regard it as a law by no means established, that "morbid poisons are not acted upon by medicinal substances as long as they continue latent." An authority of some weight in such a question, from the frequent opportunity which he has had of observation, and from his attention having been particularly turned to the latent period of some diseases, has distinctly alluded to the treatment appropriated to this period; shewing, at the least, his opinion on the subject, (Dr. Marsh, vol. v. *Dublin Hospital Reports.*) It is a question of great interest and importance, although it is one which it must be almost impossible to answer; since the only evidence of a latent period having existed is when that period has terminated. And, with regard to the second law above stated, we would ask, is not mercury antidotal to a syphilitic sore upon the glans penis, and to syphilitic inflammation of the iris; and are these tissues identical?

The following is the author's classification of diseases caused by morbid poisons:

"I. *Diseases both contagious and infectious*.—Typhus, Scarlatina, Morbilli, Variolæ, Varicella, Erysipelas, Pertussis.

"II. *Diseases simply contagious:*—Variolæ vaccinæ, Syphilis, Gonorrhœa, Hydrophobia, Pestis, Cellulitis venenata, Cellulitis farciminosa, Tinea.

"III. *Diseases neither contagious nor infectious, but depending on miasmata:*—Febris palustris, Cholera indica, Dysenteria palustris.

"This catalogue," says Dr. Williams, "might perhaps admit of some additions, as Catarrh, Influenza, Angina parotidæa, &c.; but it has been thought preferable, until the arrangement shall have received the approbation of the profession, not to introduce any disease that might give rise to controversy." (P. 23.)

It will by some be thought that diseases have been introduced into the above arrangement which may give rise to controversy; but it is not our intention now to enter into the interminable question of contagion, and we shall therefore reserve the expression of our opinion on this classification until the author's views have been explained with reference to each disease included in it. The present volume is devoted to the first class only, and typhus fever stands at its head.

Typhus. Of this disease, the author says, "there is but one simple continued fever known in this country, and that is caused by the agency of the typhoid poison." In this opinion we are disposed to coincide, as far as the knowledge which we possess of the characteristics of fevers of the present day will allow such a decision. We have witnessed continued fevers in continental hospitals, in those of London, Edinburgh, and Dublin, and in other parts; and, from the common characteristics of them all, we should regard them as the same disease. What may appear exceptions to this rule will receive considerable explanation from Dr. Williams's subsequent remarks. But, whilst making this admission, we speak only of what our own experience and much of our reading have confirmed. We must always bear in mind what is termed the epidemic constitution, as well as the fact that such an observer as Cullen has distinguished fevers of very different characters. The consequence of increased knowledge of the pathology of fevers has gradually been to diminish their recognized number; every variety of symptom or attendant circumstance having been at one time considered sufficient to justify a generic distinction. Dr. Williams has here generalized to the utmost, and we think with correctness. The existence of a typhoid poison does not admit of demonstration, but is inferred from the evident contagiousness of the disease, and from its capability of communication by fomites. The evidence is probably as strong as evidence can possibly be in favour of a point not capable of strict demonstration. Dr. Williams regards as the most probable hypothesis, that of the constant existence of the miasmata of typhus generally diffused in the atmosphere of certain countries. It is probably the hypothesis most consistent with our knowledge, but unfortunately it is only an hypothesis. This poison is subject to certain laws. It is capable of being diffused throughout the atmosphere, and, according to its intensity, of producing disease at certain distances. It lies latent a given period, gives rise to a certain series of phenomena, the course of which is modified by modes of treatment. With regard to the *mode of absorption* of the poison, Dr. Williams remarks, that "the typhoid poison, being diffusible in the atmosphere, is most easily introduced into the system by means of the mucous membranes, and more especially of the mucous membranes of the pulmonary organs. We have no direct evidence of the skin absorbing the typhoid poison; but it is so probable, that little doubt can remain on the subject." But, supposing that it was proved that in some cases the poison was absorbed, there are certain other cases, to which Dr. Williams does not allude, which would lead to the conclusion that the typhoid poison is by no means necessarily *absorbed* to produce its effects. These cases, too, are exceptions to the latent period. In the paper already alluded to, Dr. Marsh has collected several instances in which the first impression, on exposure to the effluvia of a fever patient, was on the organs of smell. He was himself so attacked with fever. When very much fatigued, he incautiously turned down the bedclothes of a fever patient, became sensible of a very disgusting smell, and felt suddenly oppressed and overwhelmed: a severe fever followed. Some curious instances of the same kind are mentioned in our last Number, p. 270, and most experienced practitioners have witnessed similar results. The inference from such cases would be rather in favour of a direct impression upon the nerves of the mucous membranes than indirectly by means of absorption. This may probably depend on the intensity of the poison. When extremely concentrated, it may produce its effects, as in the case of Dr. Marsh and Dr. Geary; when diluted, not until it has become absorbed. The period of *latency* varies much. We have referred to instances of sudden action; but "the extreme periods which the poison of typhus may lie latent vary from a few hours to a few weeks, or perhaps to a few months."

Its *coexistence with other poisons* is evident from its combination with scabies, syphilis, and erysipelas; "and the last is so frequent, that, in the London Fever Hospital, erysipelas is said to stalk from bed to bed, destroying the hope which had otherwise been entertained of the recovery of the patient."

The *pathological* changes of typhus are now so familiar to every one, that it is needless to specify their variety in kind, in degree, and in situation. But "it is on points of this intricacy that a knowledge of the general laws of poisons enables us to reconcile discrepancies so apparent, and at first sight so fatal to the hypothesis of one cause; for it is apprehended that a reference to those laws will shew that the exceptions that have been mentioned are not greater deviations from their general law than are common to the laws of poisons generally."

"Is the dose of the typhoid poison in excess, and the disease rapidly fatal, we should naturally expect, as in the case of an excessive dose of arsenic or of oxalic acid, that the morbid appearances would be either trifling or altogether wanting; while, supposing the dose to be milder, we should equally expect much more extensive marks of the action of the poison. When we observe, also, the poisons of small-pox or of scarlet fever producing their specific eruptions, sometimes on the cutis, sometimes on the mucous membranes, and not unfrequently on both, we can hardly feel surprised that the poison of typhus may sometimes attack one of the constituent parts of the same membrane, sometimes another, and occasionally different combinations of those parts. It is admitted that we cannot determine the inexplicable modification the poison must have undergone to produce these various results; but the differences that have been mentioned in no degree disprove the unity of the efficient cause, nor are greater deviations than are common to the laws of poisons generally. Every pathologist, therefore, will be prepared to admit occasional and limited differences in the seat of the disease, as also of the pathological phenomena affecting those seats in typhus fever." (P. 45.)

The outline of the morbid anatomy of typhus is very well given, and is a very valuable part of the chapter; but, as it contains nothing new, it need not detain us. We may, however, notice that later experience has shewn that the specific morbid action of typhus upon the intestinal canal is not so exclusively as was supposed upon its mucous follicles. Although this form of disease may characterize every case, one year, it may be altogether wanting in another; while, in another, every constituent part of the membrane may be occasionally affected. Dr. Williams has made a considerable omission in not having attended to the morbid condition of the blood in typhus. It would appear (and from the author's hypothesis of the poison infecting the blood, it was well deserving his notice,) that there are changes in the blood in typhus, more or less characteristic, and without an allusion to which its morbid anatomy must be incomplete; such as, for instance, the diminished, and, in some instances, exhausted coagulability, &c.

Under the head *Treatment*, is considered the question, "whether there is any mode of arresting the fever, immediately on its formation?" The evidence in favour of such an action, which has been ascribed to cold affusion is considered, and is decided negatively. The benefit derived in the cases related by Dr. Currie is ascribed, not to any power possessed by cold of *stopping* the fever, but to the agreeable effects produced on the patient's feelings by a mode of treatment introduced when the Brunonian heating system was prevalent.

"If," says Dr. Williams, "we turn from this empyrical practice of cold affusion to the great and leading doctrine of fever, which attributes this disease to the action of a morbid poison, it will be plain, if that hypothesis be correct, that cold affusion could not interrupt the course, though it might modify the symptoms. A poison circulating with the blood cannot be removed from the system by ablution of its surface. No person expects to stay the course of small-pox, of scabies, or of syphilis, by a similar application. We might therefore have predicated, *a priori*, that cold affusion could not remove from the body the poison of typhus fever, and consequently had no power to stop the course of the disease, though it might modify the symptoms." (P. 71.)

Now, with regard to typhus, much is taken for granted in the foregoing paragraph, which must be proved before it can lay much claim to our belief. It is assumed, (1) that the typhoid poison circulates with the blood; (2) that, with respect to this particular remedy (cold affusion), scabies and syphilis stand on the same ground; (3) that the effect of cold affusion is merely ablution of the surface; (4) that it is necessary to remove the poison from the body in order to stop the disease.

The first point is not proved; and we have alluded to a class of cases in which it is not impossible that fever is generated by an impression on the nerves. With respect to the second, in our ignorance of the action of cold affusion, it would be as reasonable to say that no person expects to stop ague or syphilis by sulphur, scabies by quinine, or typhus by vaccination. The effect of cold affusion is probably something far beyond mere ablution of the surface, as we may conclude from Dr. Tweedie's remarks, quoted by the author, that "it gave great alarm to the patient, and the shock was in many instances injurious, &c.;" and, with regard to the fourth question, may not the action of cold affusion, instead of removing the poison from the body, be to render it again latent? Dr. Williams believes the poison to exist in the blood during the latent stage, without manifesting any effect; so that it is scarcely fair to require proof of its being removed from the system before he will admit the possibility of the disease being stopped. We are not the advocates of cold affusion, and we believe that in this we are supported generally by the profession; but we must protest against any positive inferences being drawn from such reasoning as we have above noticed. Emetics are likewise repudiated as inefficacious in stopping the disease; and the question, as to the known existence of any antidote or specific remedy to the poison, is answered negatively. With regard to mercury, it is truly observed, that the evidence of its utility is so conflicting as "plainly to prove that its exhibition has not diminished the mortality in typhus, and that its entire abandonment would in no case lessen the chances of the patient's recovery, but in many instances might perhaps increase them." Dr. Williams has entered largely into the question of bleeding in typhus. It is unfortunate that, except in a few instances, we have little more than the authority of great names in favour of or against the practice of bleeding. But,—when Sydenham's practice was influenced by the hypothesis that fermentation was the cause of fever, by increasing the bulk of the blood, and that it required to be diminished by venæsection; when others have bled from a purely mechanical notion of the phenomena of fever; and others from the supposition that it is always symptomatic of inflammation; when, at the same time, the results are stated only in general terms, or, at least, with none of the minuteness which the importance of the case demands—we can place no reliance whatever on the mere statement of opinion as to the utility of the practice. From a careful comparison of such evidence as exists, Dr. Williams concludes that, "since the evidence against bleeding in fever so greatly outweighs that in its favour, it seems demonstrated, and by the most extensive practical experience as yet before the public on any disputed medical question, that bleeding in the cure of fever is the exception and not the rule; and, although that operation may be occasionally useful, it is only as a mode of treatment, and applicable to particular cases."

The rules of treatment, says the author, that should guide us are, first, that the disease has a course to run, and that there is a series of inflammations, modified by various circumstances, to be set up; that probably no art can prevent these inflammations; and that the general and specific actions of the poison are greatly increased by unnecessary depletion, and by whatever weakens the body.

It is considered as generally true that, although not marked by any decided symptom, in all cases in which the disease has existed but a few hours, inflammation of some part of the alimentary canal exists. In this state of the disease, a large number of experiments has been made at St. Thomas's Hospital, to determine the most beneficial modes of treatment, and with the following results:

> "Medicines were found to have little effect, either in controlling or subduing the inflammation of the intestinal canal in fever, or even in controlling the diarrhœa. It remained, therefore, to try what effects an almost purely local treatment would produce, and whether, by means of soothing the intestine, we might not moderate the inflammation; and in this manner produce, both directly and indirectly, more sanatory effects. The most effective plan is that of enemata, consisting of barley-water and of syrup of poppies." (P. 84.)

The mode of treatment is as follows:—Ten grains or a scruple of rhubarb are given, at whatever stage the patient is admitted. The bowels, having been satisfactorily emptied, an enema, consisting of a pint of barley-water together with half an ounce of syrup of poppies, is given night and morning. This simple treatment has been continued until the patient is convalescent, and has been rarely complicated by the use of any medicine. Its success has been remarkable, compared with other modes of treatment, when the fever has been of any moderate degree of intensity; so much so, that in the years immediately before the cholera, out of sixty-three cases treated thus only one died. The cholera was preceded and accompanied by a very severe fever, and the treatment by enemata was by no means so successful, one being lost out of four or five. But still it has been considered, on comparing the deaths and recoveries under other modes of treatment, that this, by enemata, was, on the whole, most successful. Many attempts have been made to render this simple mode of treatment more efficient by the topical application of leeches, or blisters, or mustard poultices; but there is no more delicate point than the interfering with the course of fever by apparently so slight a remedy as topical depletion. The occurrence of delirium after the application of leeches, or else its aggravation, is so often witnessed, as distinctly to shew that in fever, in proportion as you debilitate the patient, even in the degree perhaps necessary to save a given organ, so is the nervous system laid only the more severely under the action of the poison. But there are cases where even violent delirium is kept up, apparently from sympathy with the diseased state of the intestinal canal: these cases are not uncommon. It is difficult to determine the cases where this susceptibility exists; but, if the patient be of a full habit, and young and flushed, and the pulse 110 to 120 in the first stage of fever, the application of leeches has in general been well borne, and given relief. On the contrary, when, as in the cholera years, the patient has been full and bloated, the countenance purple, and the sputa streaked with blood, at the first onset of the disease, depletion by leeches from the abdomen, or any other part, has aggravated all the symptoms, and the patient has rapidly sunk.

As a general principle, it is considered, as shewn by a large experience, that, in mitigating the disordered states of the intestinal canal in typhus fever, there is no treatment so generally successful as that by poppy enemata, together with the application of mustard poultices to the abdomen, and occasional and moderate local bleeding. The immediate operation of the enemata is to remove from the inflamed part all that irritates it, and thus to place it under circumstances most favorable to a happy termination. The indirect effect is to produce a general glow over the whole body, to lull the brain, and to quiet the general as well as the local irritation.

In certain circumstances, the action of enemata is less beneficial: such is the case when the stomach or small intestines are the seat of disease, when the intestine is so irritable that they are immediately rejected: they are of no service when there is much meteorism, or hemorrhage from the bowels. "When the fever assumes a more complicated form, and the specific action of the poison falls on the membranes of the brain, it is probable that this inflammation has its course to run; and for a time will be but little influenced by any remedies." Moderate delirium without pain in the head will not, in one case out of twenty, require any other than the treatment by enemata; but, if severe, with injected conjunctiva, leeches may be applied: and when this form of disease is attended with a slow pulse, of forty or sixty, leeches are also required. Blisters, the author thinks, are little to be relied on. When, in cases more complicated, the lungs become affected, if the inflammation is confined to the bronchial membrane, it may generally be neglected, unless severe, when a few leeches or a blister may be required. But, should pneumonia occur, blood must be taken by cupping or from the arm; always, however, remembered the difference between such pneumonia and simple phlegmasia.

The above is an outline of Dr. Williams's mode of treatment.

"Success," it is observed, "is the only criterion in medicine; and certainly this has effected the cure of a much larger proportion of cases than any other mode I have witnessed." . . . "The simplicity also of the treatment by enemata, even supposing it not to be attended by more favorable results than any other mode, is a great recommendation; for it puts an end to the necessity of the practitioner following up symptom after symptom, all of them up to a certain period generally combated unsuccessfully: and thus prevents his being kept in a constant state of anxiety, and the patient in a state of perpetual annoyance. It is a mode of treatment that is applicable to every form of the disease, and should be adopted in every case, only omitting the syrup of poppies when the bowels become constipated. The enemata ought also to precede all local treatment of the head, for they often entirely relieve it; so that it is not right to make use of topical depletion, or other local application to that part, till after their exhibition; for otherwise we hazard the aggravation of every symptom, and often make that disease, which was before functional, now structural." (P. 93.)

The treatment by enemata is considered as affording great advantages to the system in contending against the sequelæ of fever; such as sloughing, and erysipelas.

We have already alluded to the disadvantages attending on the recommendation of a method of treatment when it rests only upon the authority of individuals; and, however high the authority of Dr. Williams, it does not except him from the application of this remark. The few cases which are appended are of little or no use in assisting our judgment. With regard to the use of enemata *alone*, it is evident that they are trustworthy only in the milder forms of fever; respecting which the eternal question arises, as to the power of spontaneous recovery. The fact of only one case in sixty-three having died at one period, that of one in four or five having subsequently died, proves almost too much as it regards the efficacy of the enemata; for the inference is, the extreme mildness of the disease in the former case. But a table containing an analysis of these sixty-three cases, minutely drawn out; together with another, similarly exhibiting those cases in which the mortality was so considerable; these, contrasted with other analyses of the results of different modes of practice employed by other physicians at the same time, both in the Hospital of St. Thomas and that of Guy, would have afforded the sort of evidence which we require, and would have been of a very satisfactory character. As records are doubtless kept of all such cases in both these establishments, may we not express a hope that, in one of his future volumes, the author will, by publishing such an analysis, remove all ground for scepticism as to the curative powers of the plan of treatment above recommended? Our own disposition is entirely to think favorably of the treatment in many cases, and to recommend its employment: to believe, also, that in others it would prove entirely inefficacious; such, for example, as those in which the use of very large quantities of vinous stimuli has appeared to act like pouring life into the patient. It is, therefore, from the existence of doubts as to the efficacy of the above simple treatment in various cases of typhus fever, that we should have rejoiced in more abundant details.

Scarlatina. The division of this disease suggested by Dr. Williams is as follows:—Scarlatina mitior (the S. anginosa of Willan); S. gravior, (corresponding to the S. maligna of Willan); S. sine eruptione, and S. sine angina, (the last being Willan's S. simplex.) In the Scarlatina sine eruptione, "the *specific* action of the poison is limited to one tissue, or to the mucous membrane of the mouth and fauces; the cutaneous exanthema being altogether wanting." There can be now no doubt of adding this to the other forms of the disease. We need not spend our time in characterizing these various forms; but our author's remarks on the dropsy, which sometimes forms a part of the disease, (one of the tertiary actions of the poison, as it is termed,) will be very acceptable to some of our readers. It usually occurs on the twenty-second or twenty-third day from the commencement of the disease, but in a very variable proportion of cases. It is generally limited to a disordered function of the cellular or serous membranes, but in a few cases it is preceded by inflammation of the pleura or peritoneum; and, in either case, languor, peevishness, constipated bowels, sickness, and vomiting, commonly precede it by several days. It very constantly begins by œdema of the face, and, in cases of considerable danger, often attacks no other part; when more general, it affects the hands rather than the feet: in a few cases, not only the face and the extremities, but the trunk and body generally, become anasarcous; and effusion also into the abdomen, head, or chest, though not commonly, is still occasionally seen. On the first appearance of the œdema, the urine is scanty and commonly turbid; and, although the quantity is small, there is a frequent desire to pass it: in this case, a pain is felt on pressing over the bladder. The urine, however, does not remain long scanty, but is secreted copiously, and continues turbid, from numerous small fibres floating in it: its chemical properties are considerably altered. In many cases, so early as the first or beginning of the second week of the fever, and sometimes later, even when no dropsy succeeds, the urine assumes a pale red or pinky colour, and resembles the water in which flesh has been washed, so that there can be no doubt of the unusual colour being caused by particles of red blood; and it has been remarked, that those who have this symptom are more slow to recover than those in whom it is wanting. But, although the red particles of the blood are absent, another constituent of the blood appears to be present in the serum, for albumen is deposited when the urine is exposed to a heat of 160°. F. The specific gravity of this urine is also lighter, varying from 1,011 to 1,017. The first appearance of œdema is usually accompanied by an acceleration of the pulse; and, if the disease terminates favorably, the œdema and accelerated pulse, in addition to the state of the urine, are the only very prominent features. But there are many sources of danger in dropsy after scarlatina; for effusion may take place into the ventricles of the brain or into the cavity of the chest, or the anasarca may become so universal and so excessive as to present a very formidable disease. When patients have died with dropsy, Mr. Hamilton conceives that he has seen the kidney, not only in the first stage of Bright's kidney, but also so far advanced in disorganization as to have been of a whitish cream or straw colour. These remarks, Dr. Williams thinks, require confirmation; for albumen is often found in the urine when the structure of the kidney is healthy; and no instance is known of recovery from dropsy with Bright's kidney, but recovery from dropsy after scarlatina is frequent.

The results obtained by the practice of bleeding, in the treatment of scarlatina, as well as by abstaining from it, are compared in the following table:

| | | | |
|---|---|---|---|
| Of 121 | treated at the | Foundling Hospital, in 1786, by bleeding | 19 died. |
| 60 | . . | London Fever Hospital, in 1829 . | 10 — |
| 181 | | | 29 |

or nearly 1 in 6.

| | | | |
|---|---|---|---|
| Whilst of 200 | treated by | mineral acids and wine, &c. . | 2 died. |
| — 160 | . | purgatives and emetics . . | 16 — |
| — 50 | . | ditto . . . . . | 3 — |
| — 45 | . | ditto . . . . . | 1 — |
| — 100 | . | mineral acids and wine . . | 3 — |
| 555 | | | 25 |

or nearly 1 in 22. From this it would appear, that the chances of recovery are diminished by the practice of bleeding in the ratio of nearly 4 to 1, as compared with the chances supposing the patient not to have been bled: but we require much more precise data before we can admit this as a fair general inference. Many modes of treatment have been suggested. The endeavour of the author is to point out the different forms of disease to which different modes are more particularly applicable, and to establish some general rule of treatment.

To the scarlatina sine angina and sine eruptione, the *nimia diligentia* caution of Sydenham is strictly applicable. The S. mitior is distinguished from the S. gravior, by presenting an inflammation of a more sthenic character. The principle of treatment here appears to be "to admit of a middle course, or of one less debilitant than by general bleeding, and less powerfully stimulant than by bark and quinine:" first, to tranquillize the stomach and to allay its inverted action, if vomiting exists, either by small doses of the sulphate of magnesia or by the effervescing draught, every four or six hours. If the tonsils have acquired the peculiar character which marks this form, (i. e. if they are enlarged, hard, and swollen,) ten or twelve leeches should be applied to the throat, which both relieve it and the accompanying cerebral disturbance. When the disease is severe, it may be necessary to repeat the leeches. The tonsils being relieved, the disease may be permitted to run its course, little influenced by medicine; saline draughts being allowed to refresh the patient. Subsequently, when the fever has declined, some tonic medicines may be desirable. In Scarlatina gravior, the tendency exists in different parts to run into mortification. A more stimulant plan of treatment is required; and, of stimulants, the author prefers wine, which requires to be continued until the patient is convalescent, and even perhaps for some time afterwards. There are cases in which it is difficult to determine, from the state of the throat, whether it be desirable to adopt the treatment by wine, or to apply leeches, or to permit the disease to run its course without interference. In these cases, it is perhaps better to err on the safe side, and to order wine, since the only inconvenience that can arise will be, that the tonsils may become swollen and tense; an occurrence easily remedied by withdrawing the wine and applying a few leeches. The principles of treatment recommended in the asthenic form of scarlatina are such, that we are surprised the author has not applied them to some forms of typhus fever. In both cases, the poison has acted by depressing to a great degree the powers of life; in one only does Dr. Williams commend the employment of vinous stimuli. In our own view of the case, the principle of treatment is identical; the degree of its applicability varying with each case.

There is nothing that need detain us in the chapter on *Measles;* and very little novelty in that on *Small Pox:* we shall therefore pass on to the following.

Erysipelas. It is in the treatment of this disease that the author considers he has met with some of the strongest illustrations of the value of his principles of practice. It will be less generally admitted than with regard to the diseases which we have already noticed, that erysipelas should be considered as both contagious and infectious; but these qualities would appear to be shown by the same kind of proof in all. It spreads through the wards of an hospital if a patient suffering with the disease is brought into them.

"The infectious nature of erysipelas has on many occasions appeared so manifest, and the danger often so imminent, that the medical officers [of St. Thomas's] considered it an imperative duty to recommend to the governors the necessity of sacrificing the houses on the North side of St. Thomas's street, for the purpose of procuring a more complete ventilation. A month seldom elapses without some new and striking instance of the infectious nature of erysipelas occurring in the older parts of the building, and it has even spread this year to the new wings; for six patients in Anne's ward have been seized with this disease subsequently to the admission of two or three erysipelatous patients into the ward." (P. 262.)

In our Fifth Number, we noticed (pp. 147, 148,) the views of Dr. Williams respecting the treatment of erysipelas. In the present volume he has entered fully into the question, still maintaining his previous views in favour of the tonic treatment. We say now as we have already said,—this is simply a question of experience. We object to all *à priori* arguments: but, if they were insisted on, we should instance Scarlatina mitior, the dropsy consequent on the action of the poison of scarlatina, pneumonia, &c., attending typhus, as justifying, according to our author's own practice, the use of depletion in a class of diseases to which, from his own *theory*, we should infer that such a practice was inapplicable. The question then occurs, whether erysipelas is, or is not, a disease similarly circumstanced to those above mentioned?

The first authority quoted, or rather misquoted, is Sydenham.

"He bled on the first day, purged the patient on the second, and again bled him on the third day; and, if these means failed, he bled twice more, interposing a day between each operation. Yet he adds, there are cases which require a very different treatment, for neither the evacuations, how frequently soever repeated, nor testaceous powders exhibited to sweeten the blood, at all avail when a noxious recrementitious matter lies deep in the skin, and cannot be removed but by such remedies as strengthen the tone of the blood, and are, consequently, proper to open the obstruction of the pores." (P. 275.)

This, however, is a misrepresentation; for the above quotation from Sydenham refers to other diseases than erysipelas, and is rendered applicable to erysipelas only by the omission of the part of the paragraph preceding that which has been above extracted. The part omitted is as follows: "I shall observe here, by the way, that, though not only this disease (i. e. erysipelas,) but the greater part of such as affect the skin, and are attended with some sort of eruption, in case they are of the chronic kind, readily yield to this method, and accordingly go off in a short time by repeated bleeding and purging; yet there are others that require a very different treatment. For neither the evacuations, &c.," (as we have just above quoted.) And again, after having mentioned medicines proper for *these* cases, (imagined by the author to be erysipelas,) Sydenham adds, "But the medicines above prescribed must by no means be used before sufficient bleeding and purging have been used, &c." We make this quotation for a double purpose: to show what really was Sydenham's opinion, and that the occupation of the mind by an hypothesis may lead it to understand a very negative as an affirmative. Cullen's evidence is in favour of bleeding, as a rule. Dr. Williams has given Mr. Lawrence's paper on Erysipelas (if, at least, he quote from the Fourteenth Vol. of the Medico-Chirurgical Transactions,) a somewhat unfair criticism. The evidence in favour of bleeding in erysipelas to be derived from that paper, is treated with little more fairness than is the opinion of Sydenham in the above-quoted misrepresentation. Why are "only seven cases of idiopathic erysipelas, and seven cases of traumatic erysipelas," spoken of, when the paper contains the histories of eighty-eight cases, many more than fourteen of which bear directly on the treatment by depletion, so strongly recommended by its author? Dupuytren's opinion was that also of Mr. Lawrence. Dr. Williams has quoted five cases as given by Dupuytren. They do not appear to be favorable examples of the advantages of such practice; but, as it is not said whence they are taken, and as Dupuytren probably, in relating them, had some other object of instruction in view than that of the effect of bleeding in erysipelas, without the context we do not know what value to put upon them. It is hardly probable that Dupuytren would have recommended a practice of this kind from such cases as those quoted. Lepelletier says that, "*in the majority of cases*, the French practitioners are against bleeding in erysipelas." Blache and Chomel say that general bleeding has often no other effect than to blanch the eruption, without *notably* abridging its duration. Bally *abstained* from bleeding or leeching, considering them "as calculated to aggravate the symptoms, to accelerate delirium, &c." Drs. Fordyce and Wells, and Heberden, disapproved of bleeding. Mr. Pearson says that it is admissible *very rarely in large towns*, and *that a repetition of it will very seldom be necessary*. Whilst erysipelas was for two years epidemic *in London*, Mr. Bromfield says that the antiphlogistic treatment was *generally* fatal, while cordials were most efficacious. Dr. Butter speaks of it as absolutely injurious, and Dr. Willan, after stating that *all ancient writers*, except Galen, *recommend bleeding*, adds, "This practice must be evidently improper in the three forms of erysipelas last described; and even in the E. phlegmonodes, it does not *always appear* necessary." To these various opinions communicated by Dr. Williams, we add the following: Biett, speaking of active forms of erysipelas, says, "Bleedings are generally indispensable;" and adds, that they should be repeated according to the increase of the symptoms. (*Sur les Mal. de la Peau.*, p. 19.) In Hutchinson's cases (Vol. V. *Med. Chir. Trans.*,) the incisions were attended with a loss of from fifteen to twenty ounces of blood; so that these cases must be regarded as affording some evidence of the advantages of moderate depletion. The cases were mostly of phlegmonous erysipelas of the lower extremities, in sailors. Of forty-one cases, it is said that not one was lost. Dr. Dobson's punctures, again, are attended with a considerable loss of blood. His practice appears to have been very successful, not only in his own hands, but in those of others; and, from our own experience, we can speak very highly of it in active cases of simple erysipelas.

Dr. Williams concludes that the weight of argument, as well as of authority is against bleeding. We are indisposed to attach any weight to the argument; and, if the authority of individuals be appealed to, it would be extremely difficult to say on which side it preponderated; or whether, indeed, the subject was one which, as it is stated, fairly admits of comparison. Before authority is allowed to have weight, it must be remembered that a disease, and not the name of a disease, is the subject of judgment. But are the sailors atDeal, under the care of Mr. Hutchinson, similarly circumstanced to the sufferers from an epidemic in London, or are the patients of Dupuytren and Dr. Williams under the same influences? From the opinions alone of these individuals, we cannot set any value upon the comparative fitness or unfitness of this or that mode of treatment. Our own conclusions would be, that the disease was subject to variations requiring corresponding variation in the treatment; that very much of the favour shewn to one mode of treatment or another depended on the natural disposition of the disease to terminate favorably, as well as on the bias of practitioners to adopt generally an antiphlogistic or stimulant system; but that it was safer as a rule to employ cautiously the former. Dr. Williams is entirely opposed to the practice of bleeding. In our Fifth Number we have stated what his practice is; we need not therefore dwell on it here.

We have already extended our notice so far that we must pass over the subject of *Hooping-cough,* which occupies the next chapter. The volume terminates with an Appendix on the advantages of the Bromide of Potassium in enlarged spleen; in illustration of which some striking cases are given.

The refutation of a law (as it was termed) of poisons, originating with Hunter and of which Adams spoke, "as well ascertained as any other in pathology," i. e. "that two actions cannot be carried on at the same time in the same part or in the same constitution," is sufficiently afforded by Dr. Williams. It is a useful lesson to those who are fond of laying down pathological laws, the existence of which often depends on imperfect acquaintance with disease, and is endangered by every addition which is made to our knowledge. Such a fate, we doubt not, awaits many of the laws contained in the present volume; which is, however, a book of very considerable ingenuity, though frequently characterized by loose and inconclusive reasoning: from the many important facts contained in it, whether the result of the author's own experience, or drawn from the experience of others, it is well worthy of a very attentive perusal.

---

